# Knee Wear Assessment: 3D Scanners Used as a Consolidated Procedure

**DOI:** 10.3390/ma13102349

**Published:** 2020-05-20

**Authors:** Saverio Affatato, Maria Cristina Valigi, Silvia Logozzo

**Affiliations:** 1 Laboratorio di Tecnologia Medica, IRCCS Istituto Ortopedico Rizzoli, Via di Barbiano, 1/10, 40136 Bologna, Italy; 2 Dipartimento di Ingegneria, Università degli Studi di Perugia, Via G. Duranti 1, 06125 Perugia, Italy; mariacristina.valigi@unipg.it (M.C.V.); sililog@hotmail.com (S.L.); 3 V-GER. S.r.l., Via G. Oberdan, 2 - 40055 Castenaso (BO), Italy

**Keywords:** biotribology, 3D optical wear measurements, 3D scanner, mobile menisci, wear maps

## Abstract

It is well known that wear occurring in polyethylene menisci is a significant clinical problem. At this regard, wear tests on biomaterials medical devices are performed in order to assess their pre-clinical performance in terms of wear, durability, resistance to fatigue, etc. The objective of this study was to assess the wear of mobile total knee polyethylene inserts after an in vitro wear test. In particular, the wear behavior of mobile bearing polyethylene knee configurations was investigated using a knee joint wear simulator. After the completion of the wear test, the polyethylene mobile menisci were analyzed through a consolidated procedure by using 3D optical scanners, in order to evaluate the 3D wear distribution on the prosthesis surface, wear depths, wear rates, amount of material loss and contact areas. The results in terms of wear rates and wear volumes were compared with results of gravimetric tests, finding equivalent achievements.

## 1. Introduction

Total knee arthroplasty (TKA) is a consolidated surgical operation in which an arthritic knee is replaced with prostheses, in order to allow patients to walk again and thus to improve their quality of life. Continue efforts have been done to improve the material properties and the design of the medical devices, and also to enhance the wear assessment methods [1,2,3,4]. 

The wear characterization of biomedical components is important, because it allows one to quantify the performance and durability of such medical devices. The gold standard for in vitro wear assessments is the gravimetric method [1]. This method, widely discussed in other works [5,6], is used to measure the amount of the mass loss and does not give other information such as surface deviations. However, the geometrical characterization of worn bearing surfaces remains necessary for understanding the wear mechanisms [7,8,9]. Recently, volumetric methods, such as coordinate measuring machine (CMM) and micro-computed tomography (micro-CT) techniques have been used as alternatives to overcome the limitations of the gravimetric method [7,10,11,12]. To quantify complex geometries such as the menisci of a total knee prosthesis (TKP), the authors proposed a validated wear assessment method and procedure involving the use of advanced 3D optical scanning techniques [13,14,15,16,17]. As widely discussed in these works, wear can be assessed, considering both the volumetric wear rates and the 3D wear distributions over the specimen’s surface. In particular, wear distribution and deformations depend on the load configuration and on the positioning adopted during the wear process. With the 3D optical scanning techniques, both the volume wear rates, and the 3D wear geometrical parameters can be measured over the entire studied surface, even if the measured object has a complex shape [14,15]. Furthermore, no contact probes are needed to perform the measurement, as it happens by using coordinate measuring machines (CMMs) [18,19], assuring faster reconstructions and examinations of the entire specimen [11]. With respect to the weighing method, the assessment of the amount of the worn volume can be correlated with the weight loss only when the exact density of the material is known and when the material is supposed to be homogenous. Otherwise, the comparison can be done on the basis of the volume and weight wear rates, which give a correct insight into the phenomenon. Micro-CT provides 3D reconstructions based on the slicing of the sample and this technique can be more useful to analyze the internal parts of the specimens, surface finish, variation of thickness and local defects [6,7,12,20].

Thus, the use and development of 3D optical digitizers in the field of biotribology, applied to biomedical devices, is a topic of great significance, as the research on cutting-edge instruments and sophisticated software tools represents an advance which will lead to enhance the scientific knowledge about all the aspects of the wear behavior of prostheses. In fact, the 3D study of a prosthesis with optical scanners and their software for 3D inspection provides one with the opportunity to check most of the wear geometrical parameters and to define the volume wear rates, the surface distribution of material loss, the volumetric loss and the contact area between the mating surfaces. An optical 3D scanner offers a simpler, cheaper and more direct method to analyze the geometry variation. 

The aim of the present study is to apply advanced 3D optical scanning techniques to assess the wear of knee mobile menisci in ultra-high-molecular-weight-polyethylene (UHMWPE). In particular, this study matches gravimetric and volumetric data in terms of wear volumes and wear rates (weight wear rate and volume wear rate) which are comparable, and assesses wear depths and contact areas during the exercise of knee prostheses, thanks to a 3D study. The knowledge of amount and position of the contact areas can give important details about how to enhance the prosthesis design to improve the wear resistance after implantation. 

## 2. Materials and Methods 

### 2.1. Wear Simulation Test

The wear behavior of TKP mobile bearing UHMWPE knees was investigated using commercially available designs. These knee prostheses were tested using a four-stations knee joint simulator under bovine calf serum as lubricant, as previously reported by Affatato and coworkers [21]. Briefly, the menisci were ethylene oxide (ETO) gas sterilized and tested in conjunction with four CoCrMo alloy femoral and tibial components (Genus mobile bearings, size #2; Adler ORTHO, Milan, Italy), consolidated by compression molding (accordingly to ISO 5834/1-2). Following a standardized protocol [22], five UHMWPE tibial components from two different production batches were pre-soaked for four weeks prior the wear tests, in order to achieve a steady level of fluid absorption, as recommended by the international standard (ISO 14243-2) [23]. After this procedure, three of these UHMWPE tibial components were chosen as test prostheses and two as check prostheses on the basis of a weighing test and of eventual shape differences visible to the naked eye. According to similarities in weight and shape, insert #1 and check #A were recognized as belonging to the same production batch and inserts #2, #3 and check #B were belonging to another production batch. Thus, the prostheses were coupled according to the following pairs: #1-#A; #2-#B; #3-#B. The achievements of this evaluation are presented in the results section, as they must be taken into account in the wear evaluation.

Then, the sample prostheses #1, #2, and #3 were tested using the knee joint simulator (Shore Western, Monrovia, CA, USA) and the other two components were used as check controls (checks #A and #B). The lubricant used was 25% (vol/vol) sterile bovine calf serum (Euroclone, Milan, Italy) balanced with deionized water and 0.2% sodium azide (Sigma Chemical, St. Louis, MO, USA) to slow down bacterial degradation. Ethylenediamine-tetra-acetic-acid (20 mmol/dm^3^) was added to minimize the precipitation of calcium phosphates on the bearing surfaces, which strongly affects friction and wear properties. The applied kinematics were in displacement control for the anterior/posterior translation, intra/extra-rotation, and flexion/extension movements. Load was applied vertically (perpendicular to the tibial tray) and oscillated in the range of 168–2600 N. All the movements replicated a simplified gait cycle according to ISO 14243-3. 

The test duration was set at 2 million cycles under a frequency of 1.0 ± 0.1 Hz. Gravimetric wear of the tibial specimens was assessed every 0.5 million cycles (Mc). At each stop for weight measurement, the cups were cleaned in an ultrasonic bath following a consolidated protocol and the ISO 14243 [21,22,23,24,25]. In particular, the mass loss was measured using a semi-microbalance (Sartorius Cubis MSE 225 S-000-DU, Goettingën, Germany), with an uncertainty of 0.01 mg and an accuracy of 0.01 mg. Each weight measurement was repeated three times and the average weight was used for calculations.

### 2.2. Preliminary Refraction And Coverage Tests 

After the wear simulation tests, the menisci were vacuum cleaned, and they were tested on optical 3D scanners to check the ability of their surface to reflect light. This ability of the material surfaces is a characteristic of great significance for optical 3D scanning procedures. In fact, in active 3D digitizers, light beams or patterns are projected onto the specimen surfaces and the whole geometries can be reconstructed by analyzing, with specific algorithms, how the surfaces reflect the light. Refracted light is a source of noise, thus, for semitransparent or transparent materials, it is necessary to apply a proper coating powder to avoid refractions. The menisci studied in this paper are made of UHMWPE, which is a semitransparent material. The preliminary reflection ability test was done by using three different 3D optical digitizers:

- Rangevision Professional Standard (Rangevision, Moscow, Russia), a tripod-mounted structured light 3D scanner for professionals.
- FreeScan X7 (Shining 3D Tech. Co., Ltd., Hangzhou, China) a 3D laser scanner for industrial metrology.
- ScanRider 1.2 (V-GER SRL, Bologna, Italy), an automated desktop structured light 3D scanner for professionals and laboratories [26,27,28].

The reflection tests were performed by using different light sources and also different light colors. All the results demonstrated that the noise level due to refractions was too high to assure reliable measurements and thus, all the menisci were treated by applying a thin layer of a 3D sublimating scanning spray (Aesub, Scanningspray Vertriebs UG, Dortmund, Germany). This kind of coating sublimates after four hours from the application and does not leave any residual on the surface. To assure the uniformity and thickness of the coverage, the coatings were applied using a painting booth, with controlled conditions. 

**Figure 1:**
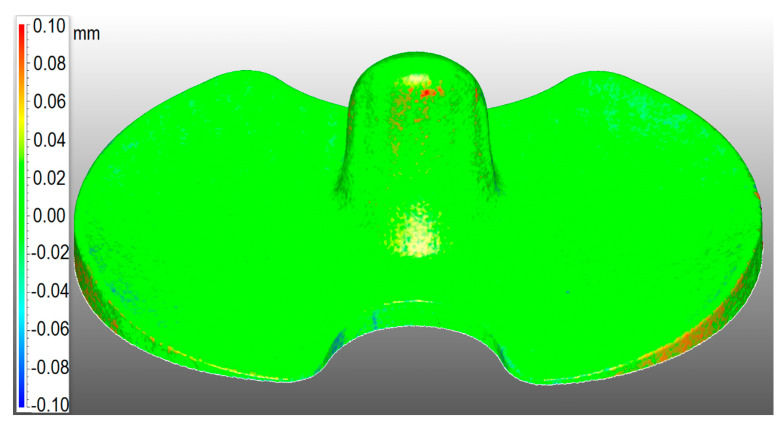
Comparison CAD-to-mesh on check A to control the uniformity of the coating.

The weight of the specimens was checked before and after the application of the anti-reflection and refraction powder with a semi-microbalance (Sartorius Cubis MSE 225 S-000-DU, Goettingín, Germany). The weighing and then the scanning of the menisci were done as soon as the coating was applied, to avoid sublimation of the spray. The results are reported in the results section. The uniformity of the coverage was checked by 3D scanning with the scanner Rangevision Professional Standard (Rangevision, Moscow, Russia), the check menisci and comparing the results with the 3D computer-aided design (CAD) project of the specimen, as shown in Figure 1. 

### 2.3. Wear Assessment Test 

**Figure 2:**
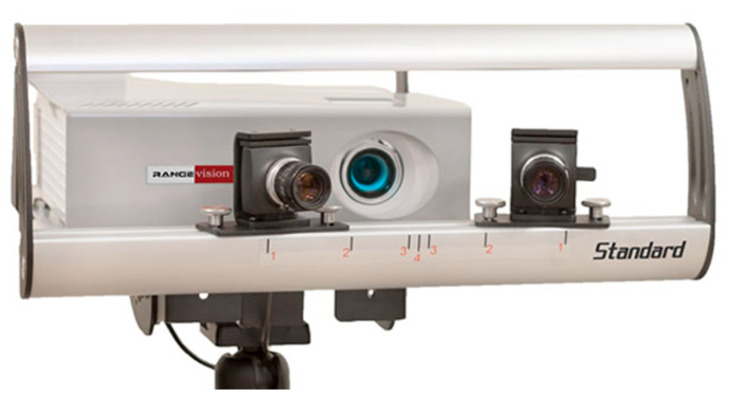
Three-dimensional digitizing instrument—Rangevision Professional Standard.

The wear assessment test was conducted throughout the 3D digitization of all the specimens. The 3D optical digitizer used in this work was Rangevision Professional Standard (Rangevision, Moscow region, Russia), a fixed structured light 3D scanner with four different scanning volumes, for objects of various sizes (Figure 2). In this paper, the 3D scanner was calibrated to be used in a scanning volume of 133 × 100 × 100 mm^3^, with a resolution of 0.1 mm and a precision of 0.03 mm. This instrument was chosen among the others used for the refraction test, because its projection system was the most powerful in terms of luminous flux and this reduced the noise. Furthermore, its resolution and precision were compatible with the application. 

The 3D digitizing procedure was automated by using a motorized turntable with a single degree of freedom (DOF) and performing scanning cycles with 10 rotations in two different initial positions of the specimens, which resulted in 20 scan frames. The samples were fixed on the turntable by using a plastic removable glue. The alignment of single scans was automatic and based on best-fit algorithms, without the use of physical markers. The software used for the scan session was Scancenter (Rangevision, Moscow region, Russia) and the software used for the registration and merge of the full 3D digital model was Geomagic Wrap (3D Systems, Inc., Rock Hill, SC, USA).

Every specimen was scanned three times to compute and take into account repeatability errors, which resulted in being negligible, as the average volumetric percentage error was 0.01%.

The 3D digital models were triangular meshes, which were further processed to be optimized for the next 3D wear characterization phase. The optimization consisted mainly of hole filling and spike removal, always preserving the original shape and dimension of the result. 

### 2.4. 3D Wear Characterization of Mobile Menisci of TKP

The wear characterization of the menisci was performed using the software Geomagic Wrap and Geomagic Control X (3D Systems, Inc., Rock Hill, SC, USA).

The 3D digital models of the worn menisci were compared with their corresponding checks, point by point, and all the deviations were registered. The method used for the wear characterization consisted of various steps. At the beginning, the reference check mesh and the worn 3D digital model were aligned and registered, according to the following pairs: #1 – #A; #2 – #B; #3 – #B. The registration was done using the check model as a reference and superimposing the worn model on top of this. The alignment method was based on the reference unworn surfaces, applying first a feature by feature registration and then a best fit global registration. The output of this procedure is a series of 3D wear maps, showing the distribution of material loss over the prosthesis surface, data about the wear depth, the friction area, volumetric loss and volumetric wear rates. 

**Figure 3:**
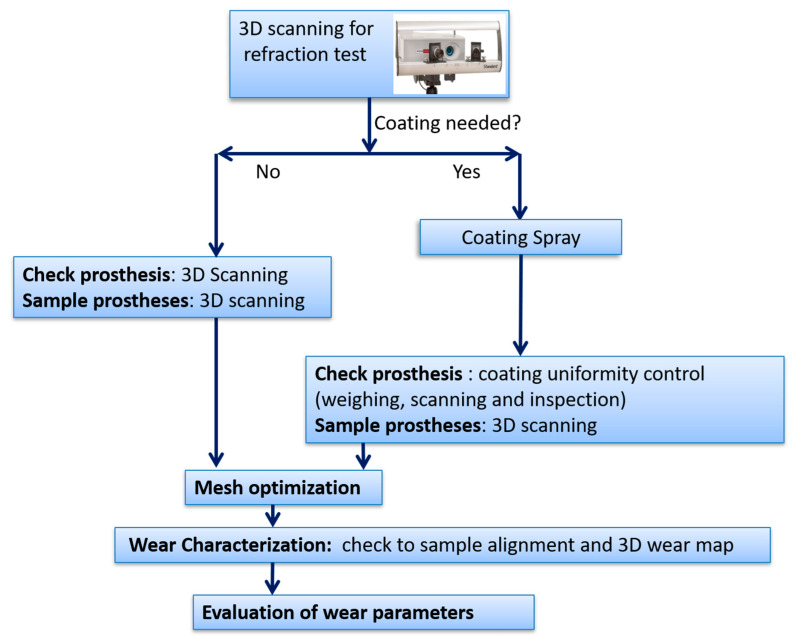
Wear characterization methodology flowchart.

Figure 3 reports the flowchart of the whole wear characterization procedure, here described, and performed after the completion of the previous steps: identification of inserts and corresponding checks and wear tests at the simulator. 

## 3. Results

**Figure 4:**
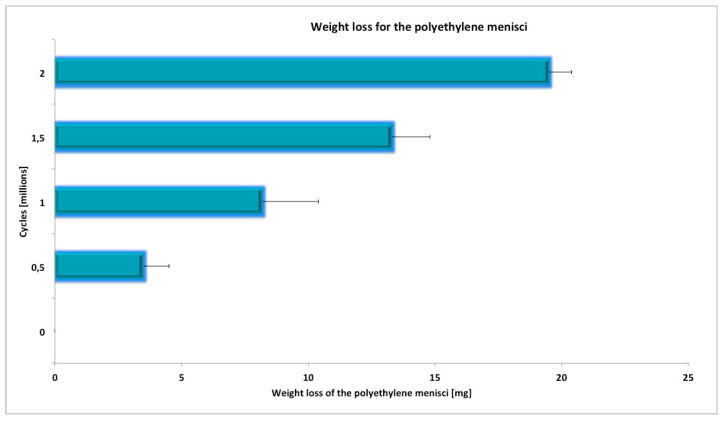
Mass loss for the mobile UHMWPE inserts size #2 tested using a knee joint simulator.

All the knee specimens completed the whole planned wear test. The average mass loss at the end of the test was shown in Figure 4. 

As the specimens could have dimensional and weight differences due to production tolerances, they were weighed before and after the wear simulation test. The results of the gravimetric measurements are showed in Table 1. The weight of the specimens was also checked before and after the application of the coating powder using the semi-microbalance (Sartorius Cubis MSE 225 S-000-DU, Goettingín, Germany). The results are reported in Table 1**,** where it can be seen that the weight percentage of the coating was very low. However, the coating weight was taken into account in the calculation of the mass wear rates, for a more precise evaluation. 

**Figure 5:**
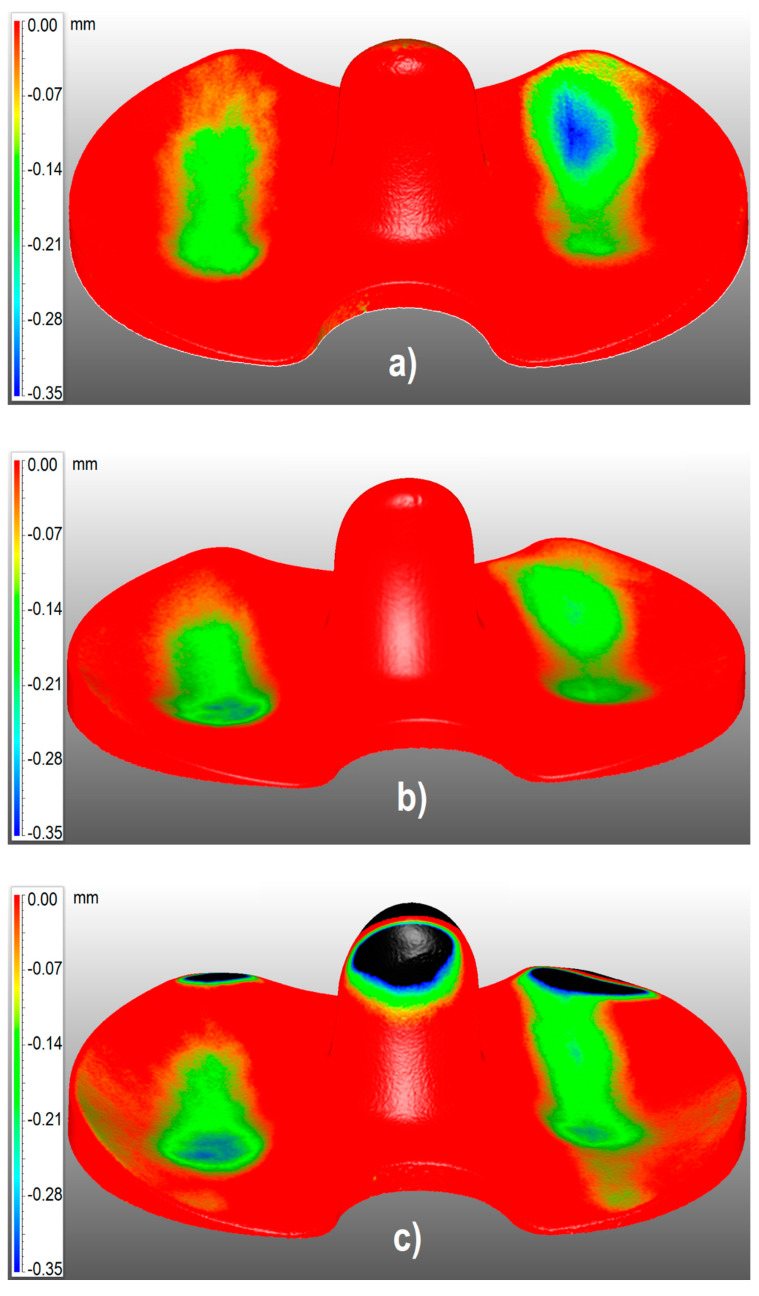
Three-dimensional wear map of meniscus #1 (**a**), #2 (**b**), #3 (**c**).

Using the 3D optical scanning measurements, the surface of all the UHMWPE inserts was characterized. In Figure 5, the 3D color maps represent the surface deviations of each meniscus from the corresponding check. The blue color represents the most worn surfaces and the red color shows the unworn zones. The black areas observed in Figure 5 represent zones where the menisci and their checks have deviations higher than 0.35 mm. As can be observed from the pictures, the presence of those areas was not due to wear, but it was caused by shape tolerances in the production process, even if the coupled prostheses belong to the same production batch. In fact, these zones did not correspond to any contact area, and for this reason, the black areas were not considered in the calculation of worn volumes and rates.

From the 3D results represented in Figure 5, further calculations of contact areas and wear rates could be performed. The volume wear rates *χ_v_* and weight wear rates *χ_w_* were evaluated, according to

(1) $$\chi_{V}=\frac{\;V_{c}-V_{m}}{V_{c}\;}\%$$

(2) $$\chi_{W}=\frac{\;(W_{c}+\Delta W_{mc}-W_{sc})-(W_{m}-W_{sm})}{\left(W_{c}+\Delta W_{mc}-W_{sc}\right)}\%$$

where *Vc* is the volume of the check, *Vm* is the volume of the meniscus, *Wm* is the weight of the meniscus, *Wc* is the weight of the corresponding check, *Wsc* is the weight of the coating on the check, *Wsm* is the weight of the coating on the meniscus, Δ*Wmc* is the initial difference of weight between the meniscus and its check.

Table 2 reports all the results of the 3D studio about the wear characterization, in terms of wear rates, wear depths and contact areas, together with the initial weight difference Δ*Wmc*.

This evaluation of Δ*Wmc* is crucial and was taken into account during the wear assessment test, because the initial weight differences affect the final results, as it can be seen from Table 2 and Equation (2). On the basis of the initial weight differences and of shape differences visible with bare eyes, it was also possible to group the available prosthesis into two different production batches, in order to couple the right pairs and make significative comparisons, as already mentioned in the materials and methods section. Thus, the prostheses were coupled according to the following pairs #1-#A; #2-#B; #3-#B. In fact, #1 and #A were recognized as belonging to the same batch, such as #2, #3, and #B belonging to another batch.

Moreover, the standard deviation of the experimental results is presented; it represents the standard deviation of the points constituting the 3D surface of each sample prosthesis, with respect to the surface of the corresponding check.

Table 3 reports the comparison of volumetric wear results, considering the gravimetric data and the 3D inspection results. The volumetric losses were calculated from the weights considering a value of 0.934 mg/mm^3^ for the density of the UHMWPE inserts [18]. 

## 4. Discussion

Given the long-term problem of PE wear, the clinicians’ interest in new approaches for the wear assessment is welcome. Prosthetic failures represent a clinical problem and eliminating or reducing wear would play a crucial role in preventing them. In this study, the application of a validated technique based on advanced 3D optical scanning was used to assess the wear of UHMWPE knee mobile inserts, in terms of 3D wear distribution on the prosthesis surface, wear depths, wear rates, wear volumes and contact areas. Data from the 3D study were compared with gravimetric results in terms of weight and volume wear rates and in terms of volumetric losses.

The results showed slight differences between the studied menisci, in terms of worn contact areas, maximum wear depth, weight, and volume wear rates. Therefore, for the meniscus #1, the wear phenomenon seemed to be more important. In fact, it presented both the biggest worn surface, volumetric loss and wear depth, giving rise to higher wear rates.

Numerous techniques have been developed to assess polyethylene wear in TKP during simulator trials and retrieval studies [1,24,25,29], but these are associated with certain limitations. The gravimetric method is the gold standard procedure to measure the mass loss of polyethylene specimens used in the orthopedic field. Nevertheless, this method often needs to be combined with volumetric 3D scanning methods. In fact, gravimetric data cannot give any information about the wear distribution over the specimen surface and about parameters like wear depth and contact areas. The gravimetric tests just define the amount of material loss in terms of weight. Furthermore, the measured weight value can be affected by errors due to changes in density, caused by liquid absorption during the storing phase. The mass loss can be correlated with the amount of the worn volume, only if the exact density of the material is known and when the material is supposed to be homogenous. Otherwise, the comparison can be done on the basis of the volume and weight wear rates. Comparing the volumetric results reported in Table 3, it is clear that the data resulting from gravimetric and 3d scanning methods are comparable. A deeper analysis of these results shows that, in most cases, the gravimetric method gives lower values of the wear volumes than the volumetric technique [18]. In fact, differential amounts of absorbed fluid in the polymeric material could result in falsely lower values of wear for the gravimetric method. Furthermore, creep can be wrongly recognized as wear when detected by 3D scanning or other volumetric techniques, like coordinate measuring machine methods and originating higher wear values. In this study, the stabilization of the test menisci before the wear evaluation was performed, in order to minimize the errors due to creep.

To the authors’ knowledge, few works can be found about the use of a digital procedure in different matters to evaluate 3D wear distribution [13,14,15,16]. The advantage of the wear assessment method based on optical 3D scanning is mainly the possibility of studying the wear phenomenon collecting many geometrical data, to evaluate most of the wear parameters and contact areas in a simple and fast way. In fact, several parameters can be evaluated, as the distribution of volumetric losses over the contact surface, the wear depths and the worn areas. Those parameters are very important in the wear characterization of prostheses, since they give a lot of information about how to modify or enhance the prostheses designs, to improve their wear resistance. 

The new recent 3D optical volumetric assessment applied in this paper offers some advantages with respect to the other volumetric wear evaluation methods, i.e. the use of micro-CT or CMMs. First of all, instruments for measuring geometry like the aforementioned apparatuses are relatively expensive if compared to an optical 3D scanner, even for metrology. Thus, researchers often continue to use the gravimetric technique as the preferred one [18].

The volumetric optical methods based on micro-CT techniques present some characteristics such as the relatively short scanning time for each specimen and the capability of analyzing the specimen structures, even locally [6]. Recently, the use of micro-CT imaging has become widespread, including for wear assessment in retrieved polyethylene prostheses [6,12,20,30]. While micro-CT applications provide 3D reconstructions based on the slicing of the sample, the use of 3D optical scanners, as was proposed in this paper, generates surface reconstructions of the object. Three-dimensional reconstructions resulting from the application of a micro-CT apparatus imply a transversal resolution between two subsequent slices equal to the slicing offset, while the resolution of the mesh resulting from an optical 3D scanning system depends only on the resolution of the device and it is the same in all directions. Micro-CT can be advantageously used to analyze the internal parts of the prostheses, the surface finish, eventual variation of thickness and local defects [6,12,20,30]. Methods based on optical 3D scanners offer simpler and cheaper procedures to analyze the geometry variation. 

With respect to the use of coordinate measuring machine techniques, the new proposed optical method avoids the use of contact probes. In literature, the use of a coordinate measuring machine is often considered as the most obvious method for measuring wear geometrical parameters [18,19], however, this technique presents some limitations. For instance, as measurements with coordinate measuring machines imply touching the object surface, on as many points as possible, for components with free-form or complex surfaces, the 3D reconstructed model could result in a surface approximation [18,19]. On the other hand, with a 3D optical scanning system, this approximation is limited to the spatial resolution of the digitizer, which can be very small. Furthermore, measurements both with CMM and micro-CT apparatuses are slow. For instance, in [11], the authors report a scanning time for UHMWPE tibial knee components of 40 min per scan for coordinate measuring machines and 90 min per scan for micro-CT systems. In this study, each scan took just 5 min.

The advantage of a contact method against an optical one is the possibility of avoiding the use of coatings for transparent or semi-transparent materials and great accuracy if applied to objects with simple and regular geometries.

In comparison to the weighing method, the 3D optical scanner warrants the direct evaluation of the distribution of the worn volumes also overcoming the uncertainty, due to the absorption of fluid during the pre-treatment of the prosthesis. Moreover, the technique used in this paper allows to characterize the entire total knee prosthesis surfaces and allows for the quantification of focal regions of deviation, including the articular and backside surfaces. 

The weight loss can also be assessed by 3D scanning, by hypothesizing the value of density, but this is an indirect evaluation, which can be advantageously replaced by the gravimetric tests. 

Therefore, this study has some limitations. First of all, a small number of specimens was used. Secondly, the use of optical 3D scanning for biomedical components can be affected by errors, due to the semi-transparency of some biomedical biomaterials, which prevents one from using light projection without the use of anti-glare coatings. Coatings can introduce errors in the wear study, so their use must be controlled and taken into account when processing the results, as has happened in the work presented in this paper. Finally, the surface wear characterization and distribution depend on the aligning method used to superimpose the worn model and the check model. This means that the aligning operations must be carefully done based on the unworn surfaces and using the same component both as a check and as a test prosthesis. In this case, the 3D scanning session should be performed before the wear simulation and after a certain amount of million cycles. Additional concerns are related to the fact that knee wear simulators are designed to perform load cycles on knee implants under conditions of walking levels, specified in ISO-14243. As demonstrated in previous works [24,25], the results observed on knee wear simulators are strongly affected by kinematics and loads applied during simulations. Future studies should be addressed to investigate the impact that kinematics and loading waveforms of more demanding daily activities [31] might have on the results of knee wear simulations. Another future work can be focused on studying the 3D wear every 0.5 million of cycles and not only at the end of the wear simulation test.

Although, in this study, both the gravimetric and optical wear characterizations were presented and applied, the results show very slight percentage differences between weight and volume wear rates, which suggests that the 3D optical method can also be used as a standalone wear assessment procedure.

The wear characterization presented in this paper represents a cornerstone both for surgeons and for prosthesis designers. Prosthesis designers can take advantages of the results by knowing the areas mainly affected by wear, in order to reinforce them. Surgeons, being aware of these results, can evaluate the best implantation techniques and positioning methods to enhance the wear resistance of inserts, trying to place the prosthesis in order to maximize the contact area and thus reducing the contact pressure and the wear depths and rates.

## 5. Conclusions

This paper summarizes the use of a new approach to quantify the entire contact zone on total knee prostheses. The results of wear assessments of the knee prostheses proposed in this study could lay the foundations for the international standardization of volumetric analysis, which is currently lacking. In conclusion, the paper proposes the application of a reliable optical protocol for the wear analysis of polyethylene knee menisci, demonstrating that the 3D optical techniques could be an important tool for wear assessment, also used as a standalone procedure. In fact, averaging the results from gravimetric and volumetric tests, the percentage discrepancies of wear rates and wear volumes were both in the order of 10%.

The technique may also be applicable for wear analysis in wear simulator studies of other polyethylene joint replacement components, such as liner or ankle medical devices.

## Figures and Tables

**Table 1:** Gravimetric results.

Gravimetric Weight	Meniscus #1	Meniscus #2	Meniscus #3	Check #A	Check #B
Meniscus weight (mg)	15,000	15,121	15,122	15,019	15,115
Coated meniscus weight *Wm*, *Wc* (mg)	15,011	15,150	15,145	15,037	15,137
Coating weight percentage (%)	0.07	0.20	0.15	0.12	0.14
Coating weight *Wsm*, *Wsc* (mg)	11	29	23	18	22

**Table 2:** Wear results.

Meniscus Number	Δ*Wmc* (mg)	Weight Wear Rate (%)	Volume Wear Rate (%)	Standard Deviation (mm)	Wear Depth (mm)	Friction Area Onto the Check Surface (mm^2^)
Check #A–Meniscus #1	‒5.31	0.162	0.169	0.0745	0.32	465
Check #B–Meniscus #2	‒19.06	0.086	0.078	0.0806	0.26	452
Check #B–Meniscus #3	‒20.50	0.089	0.100	0.1048	0.29	457

**Table 3:** Comparison of volumetric wear results.

Measurements	Meniscus #1	Meniscus #2	Meniscus #3
Gravimetric wear (mm^3^)	26.0	14.0	14.5
3D Scanning wear (mm^3^)	27.8	13.0	16.5
Average gravimetric wear (mm^3^/10^6^ cycles)	13.0	7.0	7.3
Average coordinate measuring machine (CMM) measured wear (mm^3^/10^6^ cycles)	13.9	6.5	8.3
